# Investigations into the Improvement of the Mechanical Properties of Ti-5Al-4Mo-4Cr-2Sn-2Zr Titanium Alloy by Using Low Energy Laser Peening without Coating

**DOI:** 10.3390/ma13061398

**Published:** 2020-03-19

**Authors:** Dingyuan Xue, Yang Jiao, Weifeng He, Xiaojun Shen, Yangjun Gao, Lili Wang

**Affiliations:** 1 Equipment Management and UAV Engineering College, Air Force Engineering University, Xi’an 710038, Shanxi, China; greisy2008@gmail.com (Y.G.); bluechase@163.com (L.W.); 2 Science and Technology on Plasma Dynamics Lab, Air Force Engineering University, Xi’an 710038, Shanxi, China; young_joeafeu@163.com (Y.J.); hehe_coco@163.com (W.H.); 3 Institute of Aeronautics Engine, School of Mechanical Engineering, Xi’an Jiaotong University, Xi’an 710049, Shanxi, China; 4 School of Mechanical, Aerospace and Automotive Engineering, Coventry University, Coventry CV1 5FB, UK; shenx11@coventry.ac.uk

**Keywords:** laser peening without coating, micro-hardness, compressive residual stress, fatigue strength

## Abstract

Mechanical properties, such as residual stress, micro-hardness and fatigue performance, of the Ti-5Al-4Mo-4Cr-2Sn-2Zr titanium alloy were improved via the laser peening without coating (LPwC) with a water-penetrable wavelength of 532 nm and pulse duration of 10 ns. In this paper, three kinds of laser energy, namely 85, 110 and 160 mJ were used to process the samples. The titanium alloy samples were also peened with different impact times (1, 3 or 5 impacts) at the energy of 85 mJ. The micro-hardness and residual stress distribution results provided that LPwC can introduce compressive residual stress (CRS) and also induce hardening of the target materials. Further, micro-hardness and CRS showed the increasing trends when the laser impact times increased. However, the CRS and micro-hardness decreased while the laser energy increased from 110 to 160 mJ, which was attributed to the dynamic equilibrium between the thermal and mechanical effects of LPwC. High cycle fatigue strength of the titanium alloy was significantly improved from 360 to 490.3 MPa after three impacts LPwC. The strengthening mechanism of fatigue strength subjected to LPwC was a combined effect between the laser-induced CRS and the high-density dislocations.

## 1. Introduction

Ti-5Al-4Mo-4Cr-2Sn-2Zr titanium alloy, also named as Ti-17 (service temperature ≤400 °C), has been widely used as compressor fan blades and blisks in the aero-engine due to its excellent mechanical and physical properties such as high strength-to-weight ratio, light weight and good toughness as well as excellent corrosion and wear resistance [1,2,3,4]. However, high cycle fatigue fracture is a major failure mode of these aero-engine components which always initiates from the components surface during the operation [5,6]. Accordingly, it is of great importance to enhance the surface properties of these components. In the past several decades, several traditional and novel surface treatment techniques, like shot peening (SP) [7,8], surface mechanical attribution treatment (SMAT) [9] and also laser shock peening (LSP) [10,11], have been successfully used to enhance the surface mechanical properties and refine the microstructures to improve the fatigue performance of the metallic components. Owing to the high flexibility and non-contact features of the laser, laser shock peening has evolved as a better tool to enhance the fatigue performance of the titanium alloys [12,13,14]. 

As a new technique Laser peening without coating (LPwC) is that developed by Mukai et al. in 1995 based on the conventional laser shock peening (LSP) [15]. Unlike the conventional LSP, LPwC is a process that employs low laser energy (<1 J) without any protective coatings, which can reduce the installation and operating costs and make it more viable for mass production [15,16,17]. Thus, a number of studies about the application of LPwC on the metallic materials have been conducted, such as stainless steel [18,19,20,21,22,23,24], aluminum alloys [25,26,27,28,29] as well as titanium alloys [30,31,32,33,34,35] in the past two decades. Maawad et al. [33] comparatively investigated the effect of LPwC, shot peening and ball-burnishing on the residual stress state as well as fatigue performance of three titanium alloys, namely Ti-2.5Cu, Ti-54M and LCB. Almost at the same time, Altenberger et al. [34] investigated the low and high–cycle fatigue behavior of Ti-6Al-4V at elevated temperatures after deep-rolling and LPwC. It was reported that the laser-induced high-density dislocations tangle mainly contributes to the improvement of fatigue strength. Umapathi and Swaroop [30,31] took the Ti-2.5Cu alloy as the target material and studied the mechanical properties of Ti-2.5Cu alloy before and after LPwC with different overlap rates. Later, Umapathi and Swaroop [32] investigated the effect of different laser power density and different peening times on the microstructure and residual stress as well as micro-hardness distribution of TC6 titanium alloys. Rajan et al. [35] conducted the investigation about the microstructure and found point bending fatigue performance of Ti-15V-3Al-3Cr-3Sn after LPwC. Although many previous studies have explored the fatigue performance of titanium alloys after LPwC, the specific origin of fatigue performance enhancement due to LPwC is not clear and still needs further investigation. To the best of our knowledge, the present work is the first study to report the effect of LPwC with different laser energies and impact times on the mechanical properties as well as microstructures of Ti-5Al-4Mo-4Cr-2Sn-2Zr dual phase titanium alloy. Motivated by the above observations, the influence of LPwC with different laser parameters (impact times and laser energy) on the mechanical properties, like micro-hardness and residual stress distribution as well as the high cycle fatigue strength, will be explored in this work. Furthermore, the microstructures of the samples were characterized via the XRD and TEM. This work can be utilized to optimize the laser processing parameters so as to enhance the mechanical properties of titanium alloy and thus extend the applications of titanium alloy in various fields.

## 2. Experimental Procedures

### 2.1. Materials

**Figure 1:**
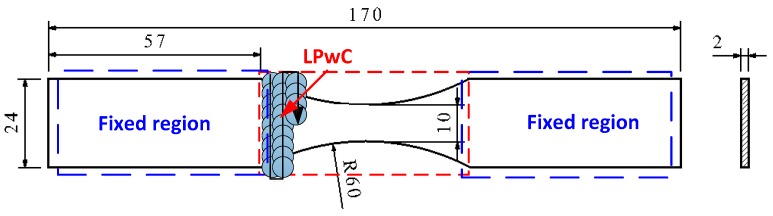
Sketch diagram of the fatigue test specimen (The red dashed region in the central arc park is the laser peening without coating (LPwC) process and the blue dashed region is the fixed area in the fatigue test).

**Figure 2:**
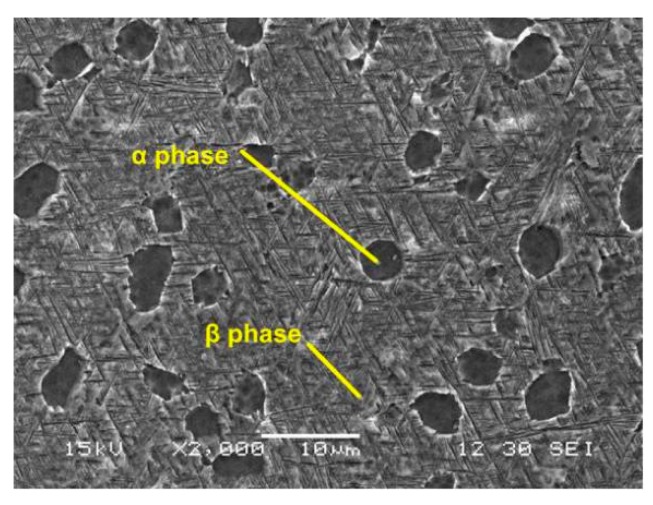
Morphology and grain size of the AC Ti-17 titanium alloy.

Ti-5Al-4Mo-4Cr-2Sn-2Zr titanium alloy master plates with the thickness of 5 mm (purchased from Baoji titanium alloy corporation, Baoji, China) were used in this study and it also has the brand of Ti-17 (it will be called as Ti-17 in the following sections). The specimens for LPwC and material characterization were cut from the as-cast (AC) Ti-17 titanium alloy plates with the dimension of 10 mm × 10 mm using the low speed electrical discharge machine (EDM). For the convenience of polishing and measurements, the square specimens were mounted on the conductive resin and left the top surface exposure. Then, they were grinded on the 800, 1200 and 2000- grid silicon carbide abrasive paper followed by the final polishing with the 1 μm diamond gel suspension on the top cloth to a mirror-like finish. According to the standard of the Aeronautical Department Standard of China (HB 5287-96 axial loading fatigue test of metal materials) the specimens for the high cycle fatigue test were manufactured. The sketch of the fatigue test specimen is shown in Figure 1. After the peening, all the samples were cleaned by the acetone in an ultrasonic cleaner to remove the debris and impurities caused during the laser processing. Ti-17 titanium alloy is a kind of α/β dual phase alloy, which consists of globular and acicular α-phase surrounded by β-phase flakes, as shown in Figure 2. This unique duplex phase microstructure leads to its excellent mechanical properties which are shown in Table 1.

### 2.2. Laser Peening without Coating

**Figure 3:**
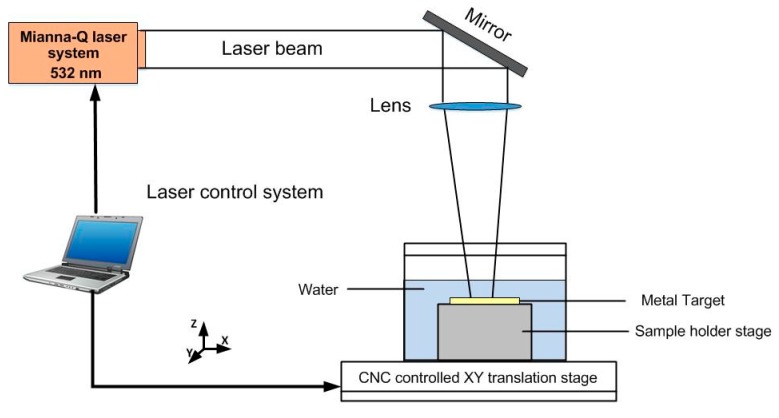
Schematic diagram of the LPwC process.

The LPwC experiment was conducted by a Q-switched Nd:YAG laser with a water-penetrable wavelength of 532 nm (Mianna Q series) which operates at 3 Hz and it can supply an maximum pulse energy of 0.8 J. The full duration half maximum (FWHM) of the laser is 10 ns and it can generate the pulsed laser with the spot size of 400 μm. Multiple pulses (1, 3, 5) and a range of laser energies (85 mJ, 110 mJ, 160 mJ) were used, and the effect of the variation of these parameters on the mechanical properties change has been investigated. The detailed laser parameters are shown in Table 2. During the laser processing, the specimens were immersed in the water tank, in which the distance between the surface of the specimen and water surface is about 1–2 mm. In addition, the specimen was fixed on the X-Y two axis translation platform and move in a zigzag scan type (as shown in Figure 1). It can be seen from Figure 3 (Schematic diagram of LPwC experimental setup) that the operation of the platform and the laser were controlled by the PC via a self-programmed software.

### 2.3. Characterization Methods

The residual stress distribution on the surface and along the depth direction of the AC and LPwCed specimens were measured via the sin2ψ-method using the Proto-LXRD stress analyzer. The electrolytic polishing layer by layer removal method was chosen for the in-depth measurement of the residual stress and the following micro-hardness test. The residual stress measurement details are shown in Table 3. The profile of hardness was obtained via using a Vickers micro-hardness tester (MVS-1000JMT2) under a load of 500 g and dwell time of 15 s, an average of five measurements was taken as the final hardness value for each depth. High cycle fatigue tests were carried out on the CCQB-100 resonant fatigue testing machine under sinusoidal waveforms with the frequency of 85 Hz and at the room temperature (22 °C). The stress ratio was set to be 0.1 and the cycle limit was 10^6^ cycles. A step-loading method was used to evaluate the fatigue results and an average of three samples under the same surface condition was used for each laser parameters.

The phase analysis of Ti-17 titanium alloy before and after LPwC was conducted via the Bruker D8 X-ray diffraction equipment using the Cu-Kα radiation (40 KV, 35 mA). The surface morphology and compositional elements were characterized by the JEOL JSM-6360LV scanning electron microscopy (SEM, JEOL, Tokyo, Japan) equipped with an energy dispersive spectrum (EDX). The fracture morphology was characterized by the SEM and the surface microstructures were characterized by the TEM-3010 transmission electron microscope (TEM, JEOL, Tokyo, Japan).

## 3. Results and Discussions

### 3.1. Analysis of Surface Morphology

**Figure 4:**
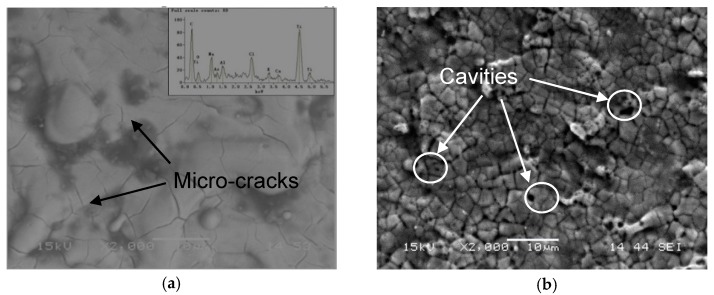

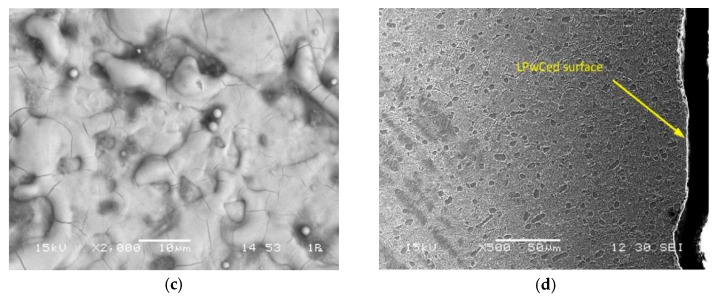
Surface SEM micrographs under the laser parameters of: (**a**) T-1 sample (**b**) T-2 sample (**c**) T-4 sample (**d**) Cross-section of T-4 sample.

LPwC is a localized thermal-mechanical process [36]. Unlike the conventional LSP, no protective coating makes the specimen directly expose to the laser irradiation which causes the laser ablation or even surface damage of the sample [37]. Therefore, minimizing the thermal damage within a certain range of laser parameters is in urgent need. In this section, scanning electron microscope (SEM) was used to characterize the surface morphology of the LPwC surface under different laser parameters, as shown in Figure 4. It can be seen that micro-cracks were generated on the surface during the LPwC. Under the intensive interaction between the pulsed laser and the sample surface, the surface molten materials were ejected away from the melt pool due to the explosive boiling. However, some of the ejected molten materials were re-solidified on the surface through water cooling and piled on the surface. During the cooling process, the molten material shrank, and some micro-cracks were generated to release the stress. The re-solidified materials should be oxide or carbide, and this has been confirmed by the EDX results shown in the inset of Figure 4a. The LPwCed surface contained 6.09 weight percentage of oxygen whereas in the AC specimen surface it was non-detectable (chemical composition of AC specimen: Ti-87.25%, Cr-4.84%, Mo-3.69%, Al-4.22%, wt.%). The oxide layer formation during LPwC should be due to the fact that the pulsed laser energy is high enough to induce the decomposition of the confinement water [18]. As a result, the decomposed oxygen reacted with the ablated surface to form the oxide layer.

As seen in Figure 4b, with the laser energy increased from 85 mJ to 110 mJ, not only micro-cracks but also the cavities were generated on the surface. Since under the higher laser energy, the explosive boiling will be more intensive, and it leads to more violent molten material ejection. Hence, more cavities were formed, and the micro-cracks were broken into smaller ones. However, with the laser impact times increased from 1 impact to 3 (shown in Figure 4a,c), there was no obvious difference of the surface morphology between them. Unlike the laser energy increasing, there were no cavities appearing on the sample surface with the increase of the laser impact times. It can be due to the fact that the interval between two successive laser impacts was long enough to let the molten material cool down but not to induce more intensive explosive boiling. These LPwC induced surface defects, such as micro-cracks and cavities, may lead to corrosion resistance deterioration of the metallic alloys [23]. In order to explore the thermal affected depth of LPwC, the cross-sectional microstructure of the T-4 specimens was examined by the SEM, as shown in Figure 4d. There was a thin white layer with the thickness of only several micrometers (less than 5 μm), which should be the ablated layer. Even though the ablated layer is only several micrometers in depth, the effect of the direct laser ablation induced surface melting on the mechanical properties still needs to be verified. In the following sections, the micro-hardness, residual stress and also high cycle fatigue performance of Ti-17 samples under different laser impact times and energy are comparatively investigated.

### 3.2. Residual Stress

It is widely accepted that the laser-induced CRS plays a significant part in the improvement of mechanical properties of metallic alloys after laser peening [38,39,40]. Laser peening without protective coating can relieve the CRS or even introduce the tensile stress on the surface due to the local melting [19]. However, some previous investigations [29,33] showed that the CRS can be induced on the surface via a good control of laser parameters. Therefore, in this section, the X-ray diffraction was used to measure the surface and in-depth residual stress distribution of the LPwCed Ti-17 titanium alloy with different laser parameters.

**Figure 5:**
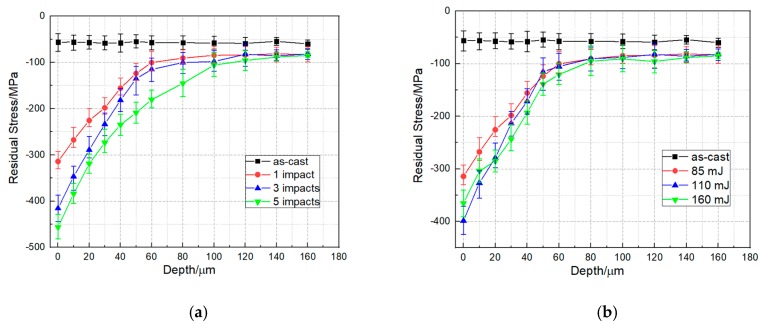
Residual stress in-depth profiles of T-17 titanium alloys under (**a**) different laser impact times and (**b**) different laser energy.

The residual stress distribution as a function of depth under different laser impact times and laser energy are shown in Figure 5a,b, respectively. The results revealed that the surface residual stress was compressive for all the specimens after LPwC. With the increase of laser impact times from 1 to 5, the surface CRS was increased from −314.2 MPa (1 impact) to −415.5 MPa (3 impact times) and then to −456.1 MPa (5 impact times). However, the residual stress decreased from −398.53 MPa to −365.3 MPa when the laser energy increased from 110 to 160 mJ. As mentioned above, the LPwC is a localized thermal-mechanical process. In particular, when the target material was irradiated by the high-power laser under the circumstance of no protective coating, some of the laser energy were absorbed to introduce CRS on the surface via the high strain-rate plastic deformation and some were for local melting. The thermal effects of the LPwC can relieve the CRS or even introduce the tensile residual stress on the sample surface. Therefore, it can be concluded that the surface residual stress distribution of target material after LPwC was determined by the combined impacts of the laser-induced plastic deformation and the laser-induced thermal effects. Under the laser energy of 85 mJ, the surface residual stress on all the samples were compressive and the CRS increased with the increase of the laser impacts, which indicated that the LPwC-induced plastic deformation dominated the process under this condition. However, when the laser energy reached 160 mJ, the residual stress became smaller than that under the lower laser energy (110 mJ), which implied that the laser-induced thermal effect tended to play a role in the residual stress distribution.

The affected depth of the specimens after LPwC with 85 mJ were 100, 120 and 160 μm, after one, three and five impacts, respectively. This suggests that increasing the laser impacts can effectively improve the affected depth. However, for the specimens treated by 85 mJ, 110 mJ and 160 mJ, the affected depth almost remained the same (about 100 μm). It also should be noted that the surface residual stress of the LPwCed specimen with 160 mJ was lower than that of the LPwCed specimens with 110 mJ, but the residual stress of the LPwCed specimen with 160 mJ started to be higher than that of the LPwCed specimens with 110 mJ from the depth of 30 μm. Therefore, it can be concluded from the above results that low laser energy with multiple laser impacts is a good parametric set to modify the residual stress distribution. 

### 3.3. Vickers Micro-Hardness

**Figure 6:**
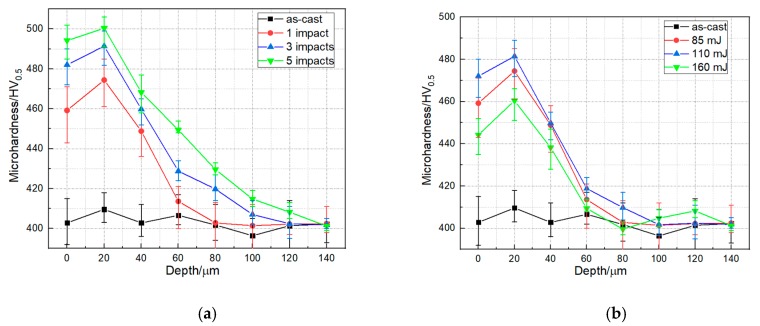
Vickers hardness in-depth profiles of T-17 titanium alloys under (**a**) different laser impacts and (**b**) different laser energies.

The components with higher hardness would have a better resistance to wear and foreign object damage to some extent [41]. Depth profile of micro-hardness under different laser impacts and laser energy are presented in Figure 6. Compared to the AC specimen, the surface micro-hardness of the LPwCed specimen were reported to increase from 402.8HV_0.5_ to 459.2 HV_0.5_, 482 HV_0.5_ and 494.2 HV_0.5_ after 1, 3, and 5 laser impacts, respectively. Similar to the residual stress distribution after LPwC with different laser energy, the increase of laser energy from 110 to 160 mJ induced the decease of the micro-hardness from 472 Hv_0.5_ to 444.2 Hv_0.5_, which was still higher than the AC specimen. It can be attributed to the role of LPwC-induced thermal effects during the higher energy LPwC process.

Along in the direction of the in-depth, it can be found that the highest hardness appeared in the subsurface layer, which was 20 μm away from the surface. Without the protective coating, the LPwC may induce the softening of the material on the surface [21,42]. That is why the highest hardness was in the subsurface but not on the surface. 

### 3.4. High Cycle Fatigue Performance

**Figure 7:**
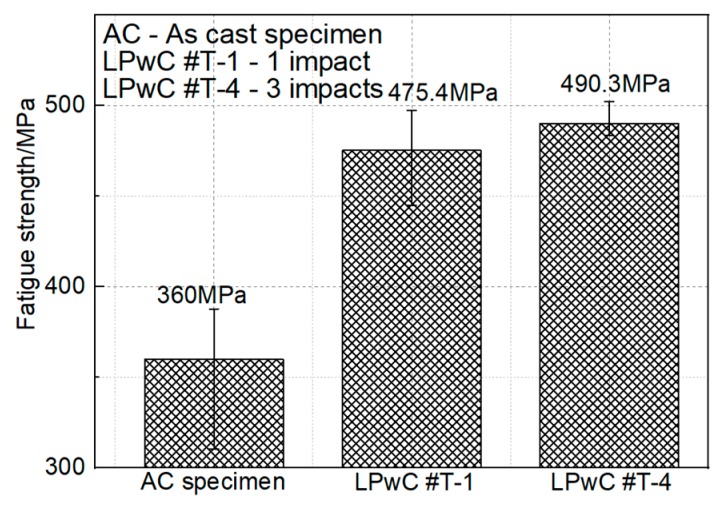
Fatigue properties comparison with different laser parameters.

**Figure 8:**
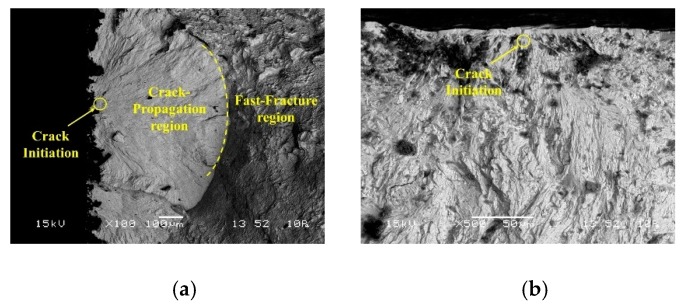

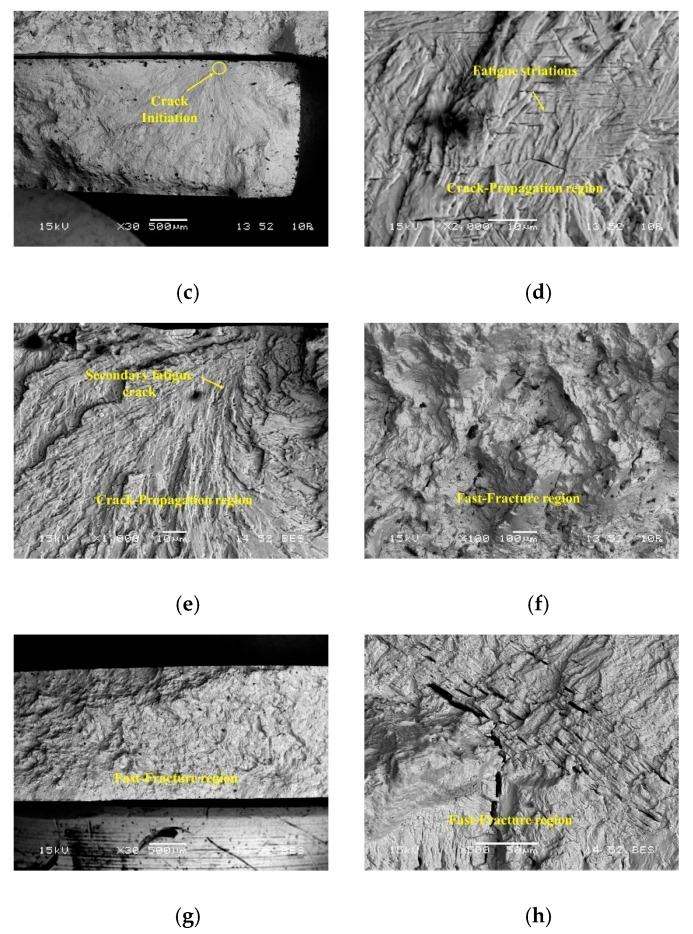
Fracture morphology of specimens after fatigue test. (**a**) AC specimen, (**b**) LPwC #T-1, (**c**) LPwC #T-4, (**d**) the crack-propagation region of the LPwC #T-1, (**e**) the crack-propagation region of the LPwC #T-4, (**f**) the final region of the AC specimen, (**g**) the final region of the LPwC #T-1, (**h**) the final region of the LPwC #T-4.

High cycle fatigue tests were carried out on the Ti-17 titanium alloy specimens in the axial loading direction under the frequency of 85 Hz and room temperature (RT) via a resonant fatigue testing machine. An average value of three specimens under the same surface condition was taken as the final result and all the results are shown in Figure 7. It has to be mentioned that these fatigue results were deduced by the step-loading method, which has been described in detail by Maxwell et al. [43]. It can be found that the fatigue strength of the original specimen was 360 MPa and the fatigue strength was improved to 475.4 MPa after LPwC with 85 mJ and 1 impact. Using the LPwC parameters of 85 mJ and 3 impacts, the fatigue strength reached 490.3 MPa. It is clear that LPwC can significantly improve the fatigue strength of Ti-17 titanium alloy with 115.4 MPa (about 32%). However, with the further increase of the laser impacts from 1 to 3, the improvement tended to be more moderate with only 130.3 MPa (about 36%). It can also demonstrate that the fatigue strength increased with the increase of laser impact times, which showed the similar trend with the CRS and micro-hardness improvement. In order to further analyze the strengthening mechanism of LPwC on the Ti-17 titanium samples, the fracture morphologies of all the samples (Figure 8) were observed via the SEM as well.

As is shown in Figure 8, the fracture surface shows a mixed morphology which consists of three sections, the crack initiation site (pointed by the arrow), the crack propagation region (the dot line included region) and the fast fracture region. Figure 8a shows all the three sections of the AC sample fracture surface, Figure 8b,c show the crack nucleation site and crack-propagation of the LPwC 1 impact and LPwC 3 impacts specimens, respectively. It can be clearly found that the crack of the AC specimen initiated from the surface (Figure 8a), but the cracks of the LPwCed specimens initiated from the subsurface, about several micrometers away from the surface (Figure 8b,c), which should be attributed to the LPwC induced CRS. Moreover, the quasi-cleavage fracture with the river-like patterns was observed on all the samples. The secondary fatigue cracks and fatigue striations, which are useful for restraining the fatigue crack propagation [16], can be observed on the LPwCed specimens. Dimples and steps can be observed within the fast-fracture region (Figure 8g,h), which implied a tendency of ductile fracture to some extent.

### 3.5. Microstructures

**Figure 9:**
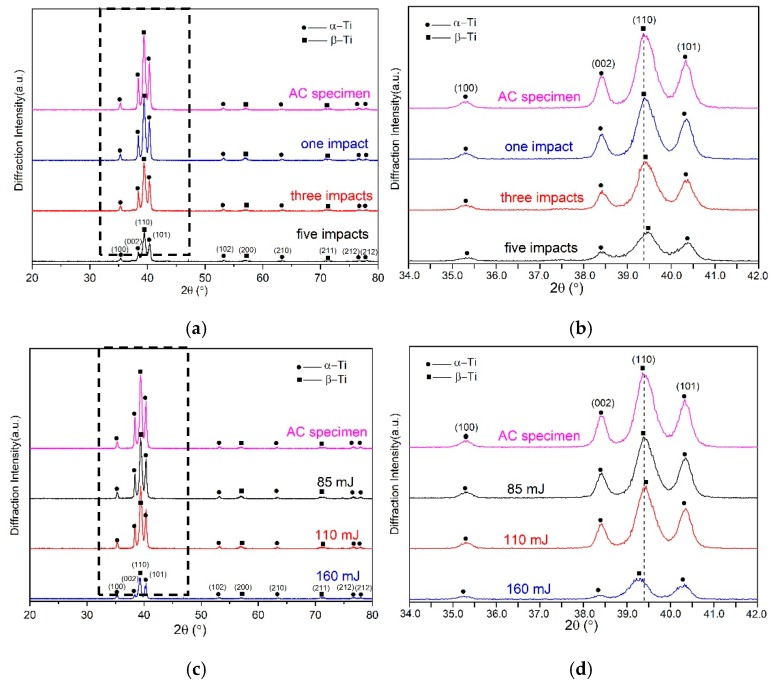
X-ray diffraction patterns of LPwCed Ti-17 titanium alloy with different laser parameters (**a**,**b**) different laser impacts; (**c**,**d**) different laser energy; (**b**,**d**) is the zoomed figure of (**a**,**c**) in the range of 34°–42°.

X-ray diffractions were conducted to do the phase analysis for the Ti-17 titanium alloy after LPwC with different laser parameters. The XRD patterns of the LPwCed samples with 1, 3, 5 impacts and 85, 110, 160 mJ are shown in Figure 9a–d are the zoomed views of Figure 9a,c in the range of 34°–42°, respectively. It is clear that the AC and LPwCed specimens predominantly consisted α-phase and β-phase (relatively lower volume fraction). Same as the laser shock peening with higher laser energy (>1 J) [14], there was no new phase generated on the surface of Ti-17 titanium alloys after the LPwC. However, the diffraction peak locations and their corresponding full width half maximum (FWHM) were changed under different laser parameters. The detailed FWHM measurements after LPwC with different laser impact times and laser energy are shown in Table 4. It can be seen that multiple pulse LPwC can induce the broadening of the crystalline peaks, and the increase of the laser impact times can lead to more obvious broadening peaks. With the raising of laser impact times, the diffraction peak location shifted to the higher angle. Nevertheless, when the laser energy increased from 85 to 160 mJ, the diffraction peak firstly moved to higher angle and then to the lower angle. This trend is similar to the residual stress and micro-hardness distribution evolution. In addition, the increase of the laser impacts and laser energy led to the diffraction intensity decrease. The broadened peak, the peak location movement and the decreased intensity should be attributed to the increase of dislocation density and the formation of refined microstructures on the surface layer of the LPwCed specimens [10,44]. In order to further explore the microstructural evolution after LPwC, higher resolution method (TEM) was used in the following section.

**Figure 10:**
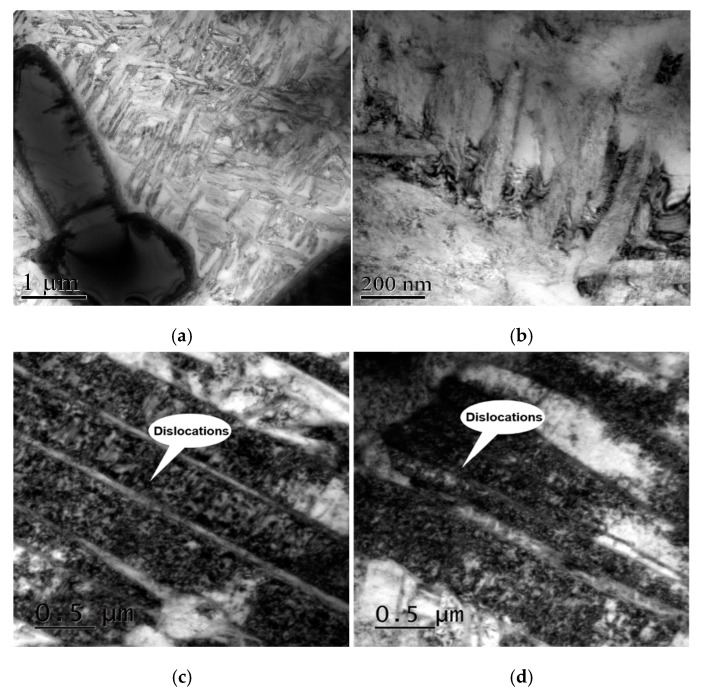
Transmission electron micrographs of Ti-17 titanium alloy (**a**,**b**) without LPwC; (**c**,**d**) LPwC with 3 laser impacts.

Figure 10 shows the TEM micrographs of the surface microstructure in the Ti-17 titanium alloy before (Figure 10a,b) and after LPwC with the laser energy of 85 mJ and 3 impacts (Figure 10c,d). It can be seen that the AC samples exhibited a low dislocation density. However, dense dislocation arrangements were observed in the LPwCed sample. It can be deduced from theses graphs that multiple pulse LPwC can produce higher density dislocations in the surface region of titanium alloy. More specifically, the high-power laser-induced shock wave is the cause for the formation of high-density dislocations. Under the effect of the shock wave, dislocations move to form the dislocation lines. With further plastic deformation, dislocation lines start to slip within the material in different directions and different slip planes [39]. As a result, more dislocations accumulate in the surface affected area and the dislocation density increases.

Based on the residual stress distribution, micro-hardness, microstructural evolution and high cycle fatigue performance, the high cycle fatigue performance of Ti-17 titanium alloy after LPwC is due to the combined effects, which can be concluded as three aspects: (1) the laser-induced CRS can reduce the working stress on the surface, so the crack initiation on the surface is prevented. Meanwhile, the increase of the dislocation density can bring in the improvement of the yield strength of the material [45]. Therefore, it is more difficult for cracks to initiate on the LPwCed sample than the AC sample; (2) the CRS can significantly increase the closing force of microscopic cracks and retard the crack propagation via blocking and swerving [46]; (3) high density dislocations can induce more grain boundaries which can restrain the slip deformation and plastic flow for crack growth.

## 4. Conclusions

In this work, the effect of LPwC, with different laser impact times and laser energy, on the mechanical behaviors and microstructural evolution of Ti-17 titanium alloy were addressed. The main conclusions from the present study can be summarized as follows:

- A large value of CRS (−314.2MPa) was introduced on the titanium alloy after the LPwC. The increase of laser impacts can introduce larger CRS, but the higher laser energy can deteriorate the CRS, which was proposed to be due to the thermal effects of LPwC.
- Micro-hardness measurements showed that 22.7% improvement of hardness can be achieved after LPwC with 5 impacts. Softening with higher energy (160 mJ) in single peening was observed. In accordance with micro-harness and residuals stress results, it can be concluded LPwC with lower energy and multiple laser impacts is an effective to enhance the mechanical properties of Ti-17 titanium alloy.
- XRD studies and TEM observations revealed that high density dislocations were introduced by the LPwC on the titanium alloy.
- High cycle fatigue tests showed that LPwC can effectively improve the fatigue strength from 360 MPa to 475.4 MPa (1 impact) and then 490.3 MPa (3 impacts), which is attributed to the LPwC-induced CRS and high-density dislocations.

## Figures and Tables

**Table 1:** Mechanical properties of the Ti-17 titanium alloy.

Material	Density (g/cm^3^)	Ultimate Tensile Strength (MPa)	Yield Strength 0.2% (MPa)	Elongation (%)	Hardness HRC
Ti-17	4.65	1120	1030	15	40

**Table 2:** Laser processing parameters for Ti-17 titanium alloy.

Specimen Number	Wavelength (nm)	Spot Diameter (μm)	Pulse Duration (ns)	Repetition Rate (Hz)	Overlappingrate (%)	Pulse Energy (mJ)	Laser Impacts
T-1	532	400	10	3	60%	85	1
T-2						110	1
T-3						160	1
T-4						85	3
T-5						85	5
T-6	As-cast (AC)						

**Table 3:** Parameters for the residual stress measurement.

Item	Description
Radiation	Cu-kα (λ = 1.540562 nm)
Voltage and current	30.0 KV and 10.0 mA
X-ray beam size	2 mm
2θ range	137–145°
electro-polishing solution	10 vol% perchloric acid and 90 vol% methanol

**Table 4:** Full duration half maximum (FWHM) of different crystal planes on Ti-17 titanium alloys before and after LPwC.

Specimen	(100)	(002)	(110)	(101)
T-1	0.28°	0.26°	0.46°	0.36°
T-2	0.28°	0.26°	0.48°	0.34°
T-3	0.36°	0.34°	0.48°	0.38°
T-4	0.36°	0.28°	0.44°	0.36°
T-5	0.38°	0.34°	0.58°	0.48°
T-6	0.28°	0.26°	0.46°	0.32°
